# Investigating and Developing a Practical Domestic-Medication System of Public Health for Chinese Family

**DOI:** 10.3390/ijerph20021060

**Published:** 2023-01-06

**Authors:** Chen Xu, Yunyi Zhang, Yun Chen, Chao Gong

**Affiliations:** School of Design & Arts, Beijing Institute of Technology, Beijing 100081, China

**Keywords:** stocked medicines, domestic-medication system, Chinese family

## Abstract

(1) Background: The main research aim of this paper is to investigate the commonly stocked medicines in Chinese households. Firstly, a large number of questionnaires were collected to uncover the problem: most Chinese families have the habit of stocking their family medicine boxes. However, there is a lack of a standardized, systematic, and scientific list of household medicine stockpiles. As a result, there are major problems in stocking medicines in households: (1) There is little connection between the type and quantity of medicines stocked and real life; (2) The expiration date of medicines leads to misuse and waste of medicines; (3) The existing list of medicines can provide little help. (2) Methods: The preliminary drug stock list was summarized through case studies; the authenticity of the questions and the credibility of the list were verified through interviews; the number of different types of drugs and the relationship between the resident’s perception of the importance of drugs and their frequency of use was determined through questionnaires; the authenticity of the list was verified through interviews with senior doctors. (3) Results: We finally composed a scientific and practical list of common household medicines, developed a practical domestic-medication system for Chinese families, and conducted validation studies, which received the approval of senior doctors. (4) Conclusions: (1) Chinese families need to prepare medicines according to the actual composition of the family; (2) Chinese families need a scientific and systematic list of commonly prepared medicines; and (3) in addition to the types of medicines, it is also necessary to consider the number of individual types of medicines to be stocked.

## 1. Introduction

About 78.6% of Chinese households have a family medicine cabinet [1], but the types of medicines stocked are not very relevant to the actual needs of life [2]. Especially in the post-epidemic era, in the face of regular epidemic management, the family medicine cabinet is often unable to fully cope with unexpected situations in life. The number of drug reserves is not completely consistent with the frequency and amount of use, which brings about the situation that many families want to use certain drugs, but the drugs stored at home have expired. Moreover, because more than 80% of the families do not have the habit of regularly cleaning the medicine boxes, the country produces about 15,000 tons of expired drugs in a year [1]. At present, China has not yet established a mature, scientific, and perfect recycling system for expired drugs [3,4,5], and such a serious waste of drugs is not conducive to the green and sustainable development of the environment. Therefore, solving the problem of expired drugs in household stockpiles is urgent. Existing studies on stocked medicines are based on the U.S. socio-medical situation [6,7,8,9] and are relatively old, with insufficient guidance for the 2020s after the outbreak of the COVID epidemic.

There is a wide range of domestic medicine lists in China, including the recommended list of household emergency supplies issued by the national emergency management department and specific recommendations based on that list [10], as well as recommendations from major authoritative health organizations and experienced physicians. These lists are similar in content but differ in detail, which causes some cognitive confusion for users. At the same time, these lists do not indicate the amount of each drug to be stocked, nor do they list the details according to the age [11,12,13], region [14,15,16], number, and structure [17,18,19] of the family and other aspects [20,21]. Therefore, we found that such a generalized list is of little reference for family practice.

In addition to this, many people tend to ignore the harmful effects of expired drugs on the body [4]. In fact, expired drugs are not only less effective or ineffective, but also may cause drug resistance, allergy, shock, and other adverse reactions [22]. For example, expired sulfonamides and penicillins are prone to allergies and shock; expired tetracycline contains degradation products that are tens of times more toxic than tetracycline and can lead to damage to kidney tubular function; nitroglycerin, which is used for angina first aid, is highly volatile and can easily fail due to improper storage, which will reduce its role in first aid, etc. If the storage method is changed, such as put in a high-temperature environment, humid environment, the cap is opened, etc., it will lead to moisture absorption, dehydration, mold, and changes in the chemical composition or structure of the drug, resulting in some decomposition products of unknown effect, in this case the expiration date of the drug can only be used as a reference. If patients continue to take such expired drugs, it will not only delay the treatment of diseases, but also produce acute (slow) toxicity and side effects, which may cause unnecessary damage to the human body.

Based on the current problem of expired family medicine storage in China and the situation that it is difficult to obtain a reference from the existing family medicine storage list [23,24], we hope to propose a new catalog of household medicine stockpile list and provide details of medicine stockpile according to the different ages, regions, and family structures of the residents in China. Therefore, we first analyzed the existing drug list to summarize the types of drugs in the family stockpile; then, we studied the specific situation of the family stockpile through interviews and questionnaires, and sorted out a set of drug lists according to the collected data to meet the actual needs; finally, our list was revised and optimized through interviews with senior doctors to obtain a list of household stockpiled drugs based on the current situation of drug use in Chinese households and to propose sub-tables that differ according to the age of the residents and the number of people living with them, as well as conditions when the household contain the old and the children.

## 2. Materials and Methods

### 2.1. General Description

The study was divided into three steps: 1. Analysis of the existing drug list: summarize the basic categories of domestic reserve drugs through case analysis; 2. Familie’s actual drug reserve research: Through the questionnaire and interview, to understand the families’ actual drug list. Then, developing a new practical home-drug system; 3. Finally, the built system was verified and reinspected according to the verification results.

### 2.2. Analysis of the Existing Drug List

By analyzing the classification of household drug storage by various major network platforms, we could understand the criteria and basis of their drug list classification to classify and formulate more scientific and popular drug lists.

#### 2.2.1. Sample Selection

Four popular existing drug lists in China were selected as samples for analysis. They are: the drug list of Popular Science China, Dingxiang Doctor, PSM Pharmaceutical Shield Charity, and Xinhuanet. Then, the lists were sorted out the list. The drug information and classification, and the advantages and disadvantages of each sample are summarized in Table 1.

#### 2.2.2. Conclusion and the Next Step

According to the classification items of household drugs on major medical science websites, we formulated our questionnaires.

First of all, most list for cold, fever/pain relief, dermatitis, and diarrhea/diarrhea symptoms classification and give the recommended storage drugs, so in the questionnaire, we asked whether the people store the drugs, and the type of storage they used to determine the number of our drug list of all kinds of conventional drugs. At the same time, we found that the main website for drug classification is too generic, not for a specific age or region, and the number of people to put forward the classification and quantity of drugs, so the questionnaire addressed these three elements to investigate the relationship between them and the drug use and storage, so that we could formulate a more standard and scientific form.

### 2.3. Investigation of Actual Family Drug Reserve: Questionnaire Study and Interview Study

The second step of this work focused on families’ actual drug reserve research. Interviews and questionnaires were used to investigate families’ actual drug list.

#### 2.3.1. Interview

##### Main Question Setting

A semi-structured interview was employed in this step. Based on the previous case analysis, we designed the interview questions around the obtained drugs (Table 2).

##### Participant Recruitment

We selected 10 interviewees from different families, ages, and regions to determine the initial direction of our questionnaire design by integrating their content.

(1) Setting up

Each interviewee received an introduction email that included the interview questions list and an information sheet (the description of interview aim, method, and the use of data). The email also linked to a self-booking system where the participant could easily select their interview time.

(2) Introduction

Each interview consisted of two personnel who are the interviewee and the interviewer (the researcher). The interviewer showed the information sheet and briefly summarized the interviewee’s study before the primary interview started.

(3) Agreement signature

A consent form was provided, which presented eight relevant clauses about the agreement of participating in this study. Each interviewee was required to read and sign. Otherwise, the interview would not be continued.

(4) The main body of the interview

The interview followed 11 questions (Table 2). Each interview was audio-recorded with each interviewee’s permission.

##### Data Collection and Analysis

After collecting and integrating the interview results, we summarized and integrated 10 questionnaires. By analyzing the storage and use of different drugs in various types of families, we roughly prepared our questionnaires and obtained more universal results through the questionnaire survey.

#### 2.3.2. Questionnaire Survey

##### Questionnaire Design

**Figure 1:**
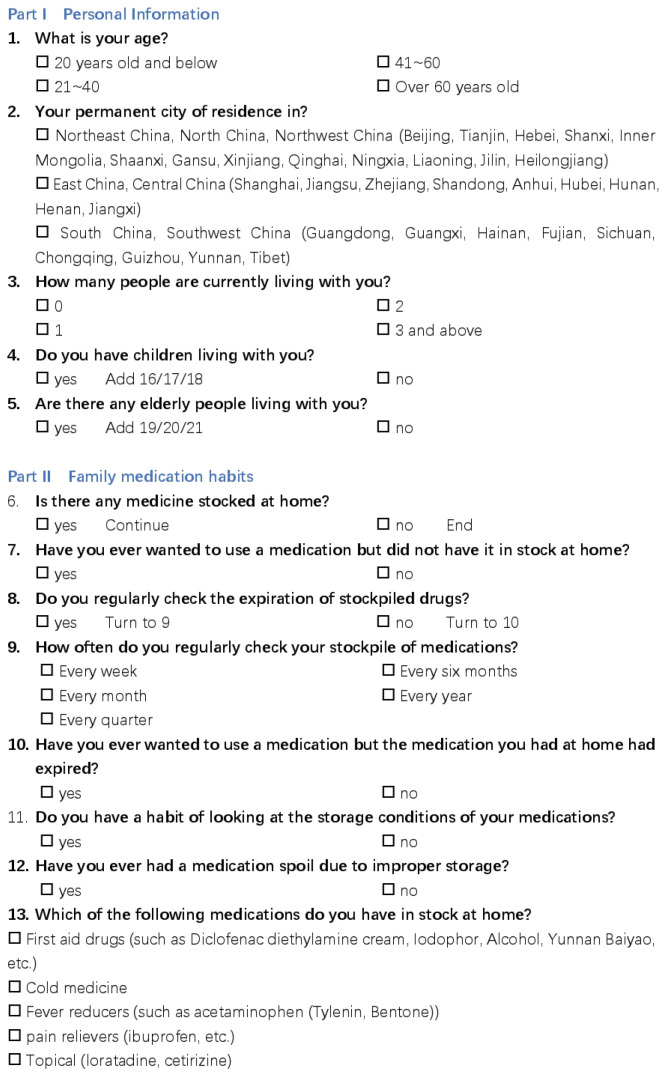
The detail of the questionnaire.

**Figure 2:**
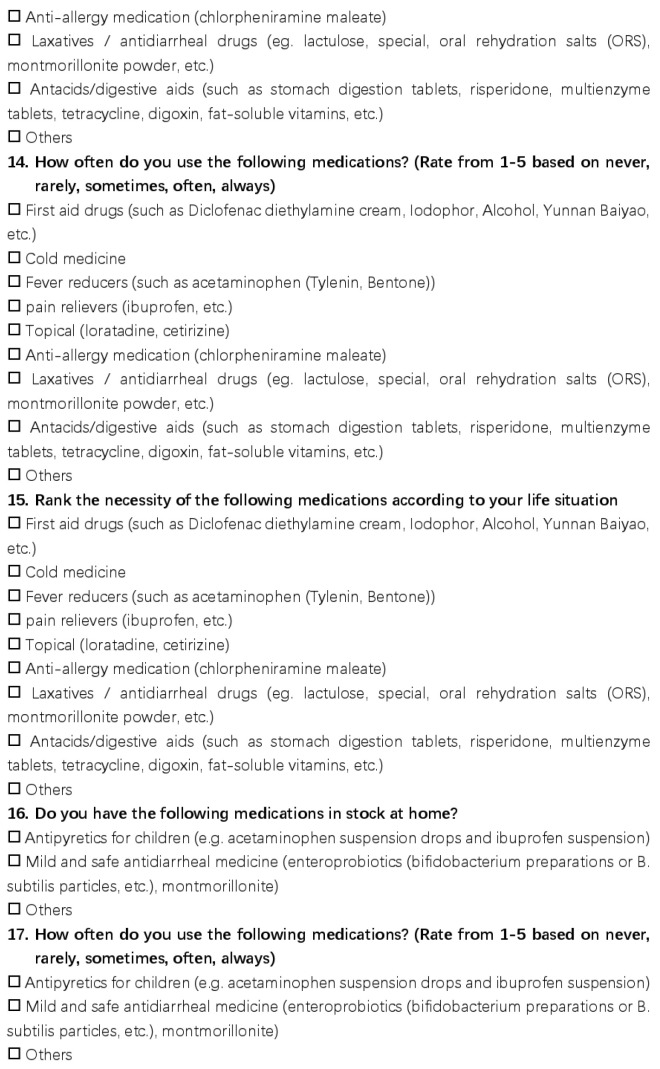
The detail of the questionnaire.

**Figure 3:**
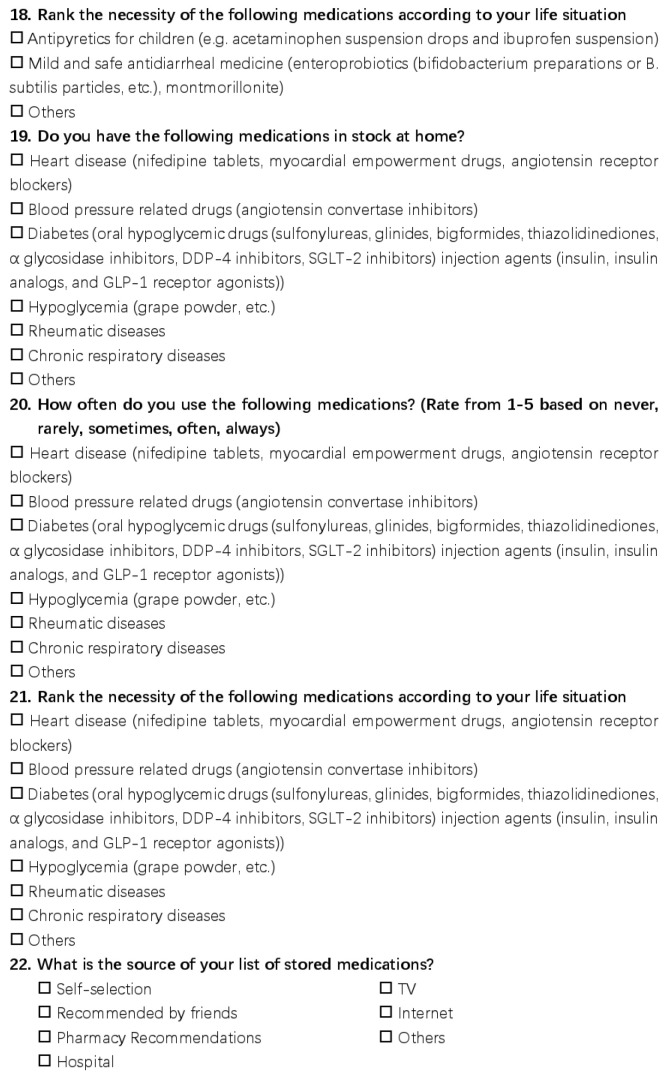
The detail of the questionnaire.

This questionnaire had a total of 21 questions and was distributed in the form of an online questionnaire, the details of which are shown in Figure 1, Figure 2 and Figure 3. The questions asked about the basic family situation and the storage, frequency, and importance of the medications we list.

##### Participant Recruitment

A total of 526 questionnaires were distributed and 526 questionnaires were collected, of which 476 were valid.

##### Data Collection and Analysis

According to the data collected, we used the frequency of all kinds of drugs in the ranking; first, we recovered 476 valid questionnaire for data processing, ranking linear regression frequency and importance. Based on the importance of the drugs determined from the calculation, and in accordance with the expected results, some drugs’ frequency of use is low, indicating that people generally pay little attention to such drugs and also illustrating the importance of developing a more scientific and universal table.

## 3. Results

### 3.1. Case Study

Through the analysis of different lists, based on the method of forming hospital drug lists, we summarized the types of drugs that are stocked at home in Table 3, Table 4 and Table 5.

### 3.2. Interview

**Figure 4:**
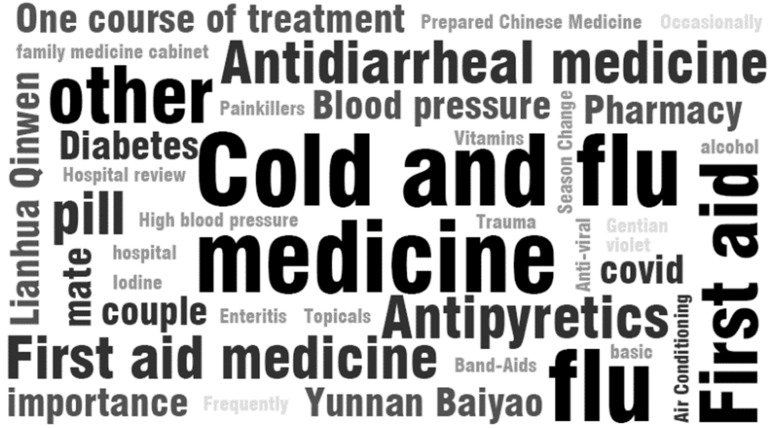
Word cloud generated based on interview results.

We found that many families were in the situation of “wanting to use a certain medicine but the medicine stocked at home has expired” through the interviews and research. The word cloud based on the interview content is shown in Figure 4, and the content analysis is shown in Table 6. According to Figure 4, the frequency with which respondents mentioned these elements broadly demonstrates the importance of these medicines to a family.

### 3.3. Questionnaire

A total of 526 questionnaires were distributed, and 476 valid questionnaires were collected.

#### 3.3.1. Data Summaries

**Figure 5:**
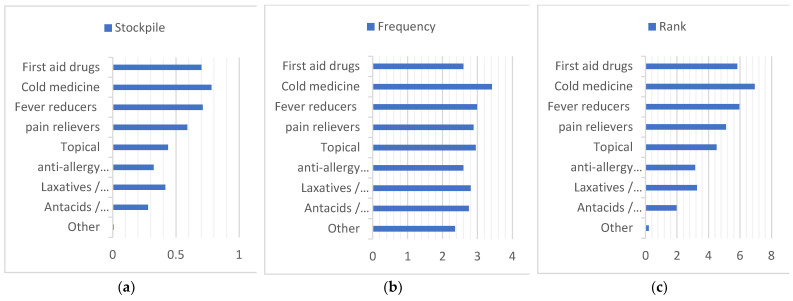
Summary of the overall drug stockpile, frequency of drug use, and ranking of drug importance. (**a**) Stockpile, (**b**) Frequency, (**c**) Rank.

**Figure 6:**
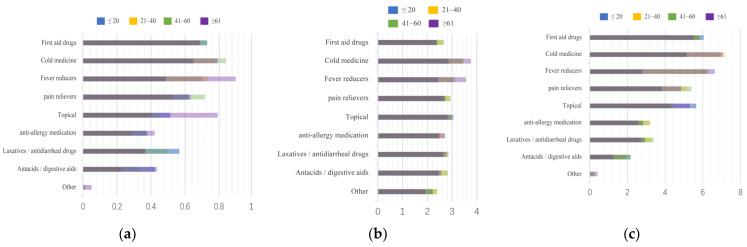
Summary of drug stockpiling, frequency of drug use, and ranking of drug importance by age. (**a**) Stockpile, (**b**) Frequency, (**c**) Rank.

**Figure 7:**
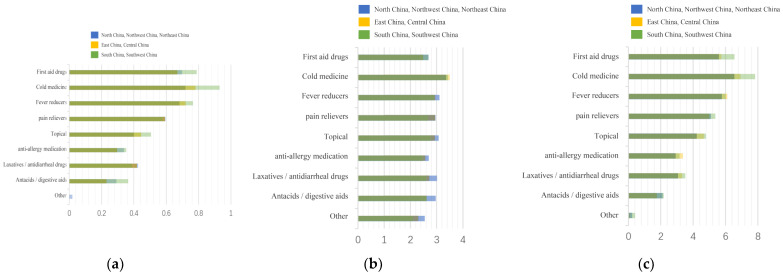
Summary of drug stockpiles, frequency of drug use, and ranking of drug importance in different regions. (**a**) Stockpile, (**b**) Frequency, (**c**) Rank.

**Figure 8:**
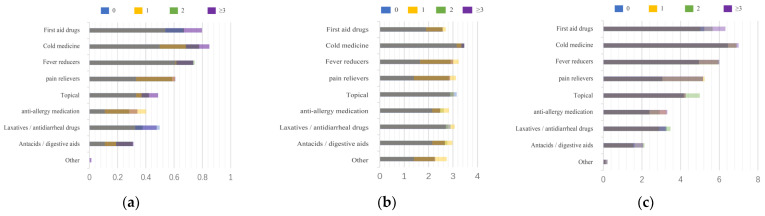
Summary of drug stockpiling, frequency of drug use, and ranking of drug importance for different number of people living together. (**a**) Stockpile, (**b**) Frequency, (**c**) Rank.

**Figure 9:**
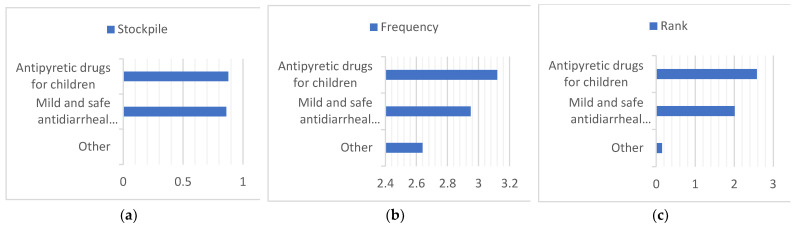
Summary of the child-specific medication stockpile, frequency of medication use, and ranking of medication importance for the child’s family. (**a**) Stockpile, (**b**) Frequency, (**c**) Rank.

**Figure 10:**
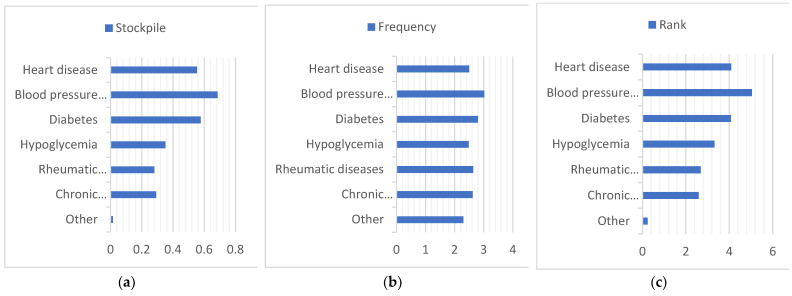
Summary of medication stockpiling, frequency of medication use, and ranking of importance of medication for elderly households with elderly members. (**a**) **Stockpile**, (**b**) Frequency, (**c**) Rank.

First, we summarize all the data in Figure 5, and to facilitate the comparison of different regions, ages, and cohabitants, we stacked the data obtained on a graph in Figure 6, Figure 7 and Figure 8. Households with children and households with elderly members are listed separately, as in Figure 9 and Figure 10.

#### 3.3.2. Data Regression Processing

As Table 7 and Table 8 show that, the value of 1.788 is slightly less than 2.0, which is slightly correlated but does not affect it. R^2^ = 0.572: The ranking of people’s importance of medicines can affect 57.2% of people’s frequency of using medicines, which means that establishing the correct concept of the importance of medicines can improve people’s scientific perception of the storage and use of medicines to a certain extent.

Significance 0.018 > 0.005 so people’s ranking of the degree of drug use cannot significantly impress the frequency of drug use, and the impression coefficient is 0.105 for a positive, positive impression.

**Figure 11:**
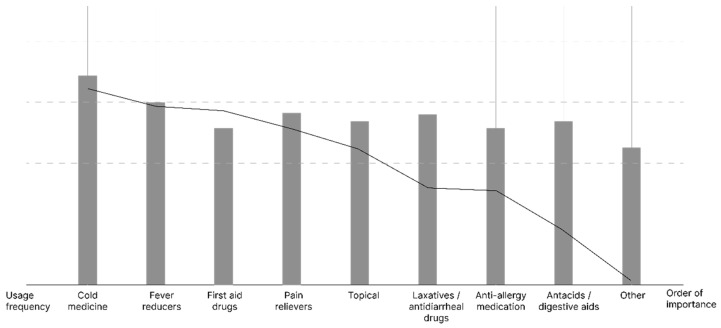
Frequency and importance of different types of drugs correlation analysis chart.

From the analysis results (Figure 11), it can be seen that there is no significant linear relationship between the frequency of use and the ranking of the importance of drugs, and it can be known that most households do not pay much attention to the understanding of various types of drugs, which may lead to unreasonable and irregular storage methods and stockpiles of drugs.

### 3.4. Preliminary Establishment of a Domestic-Medication System

**Figure 12:**
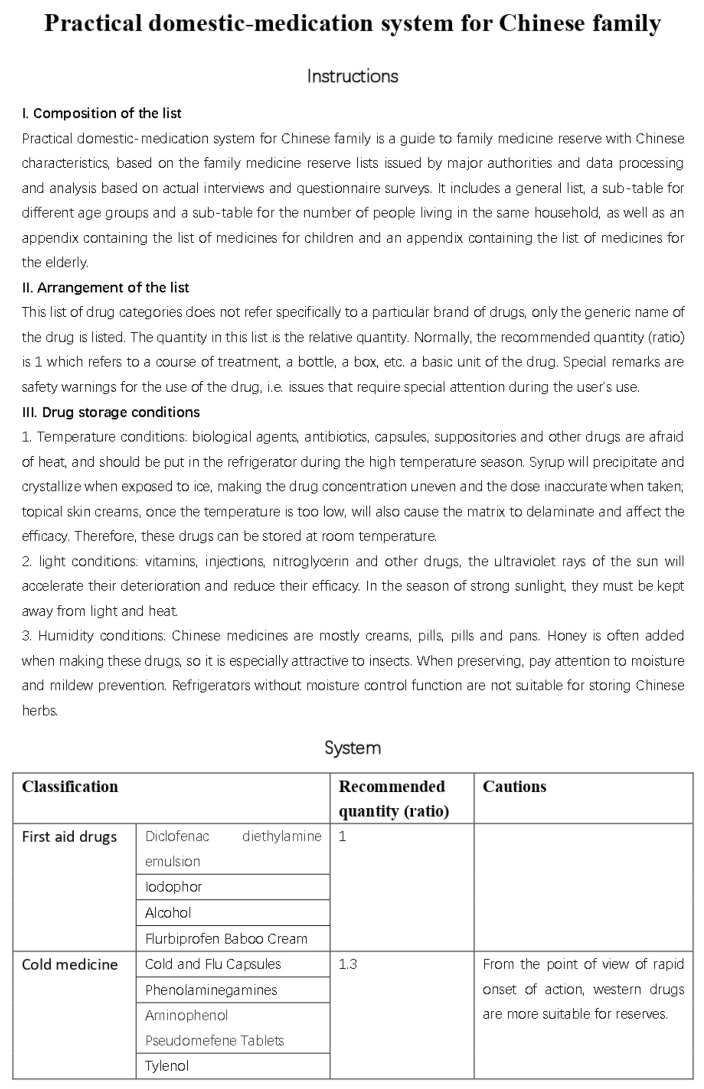
The initial version of the system.

**Figure 13:**
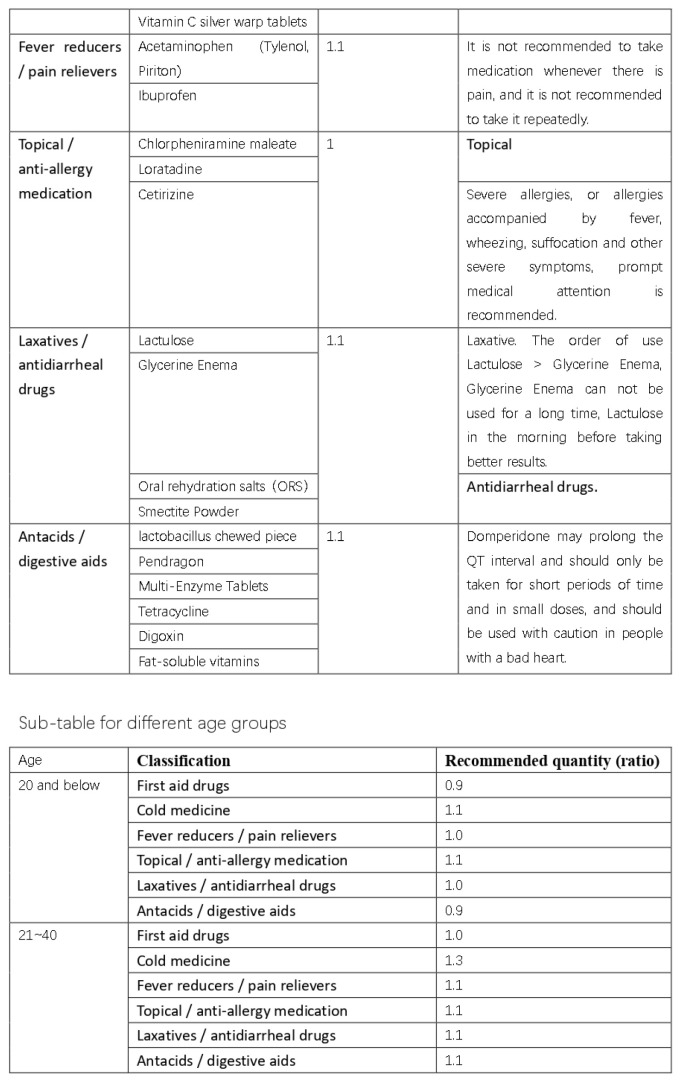
The initial version of the system.

**Figure 14:**
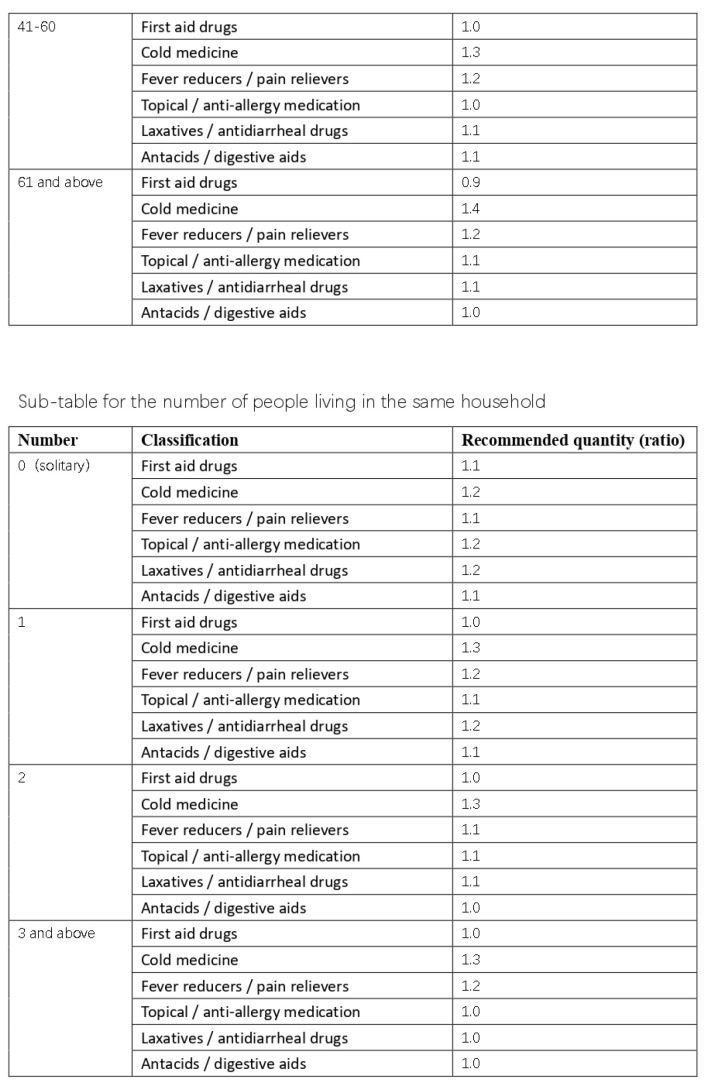
The initial version of the system.

**Figure 15:**
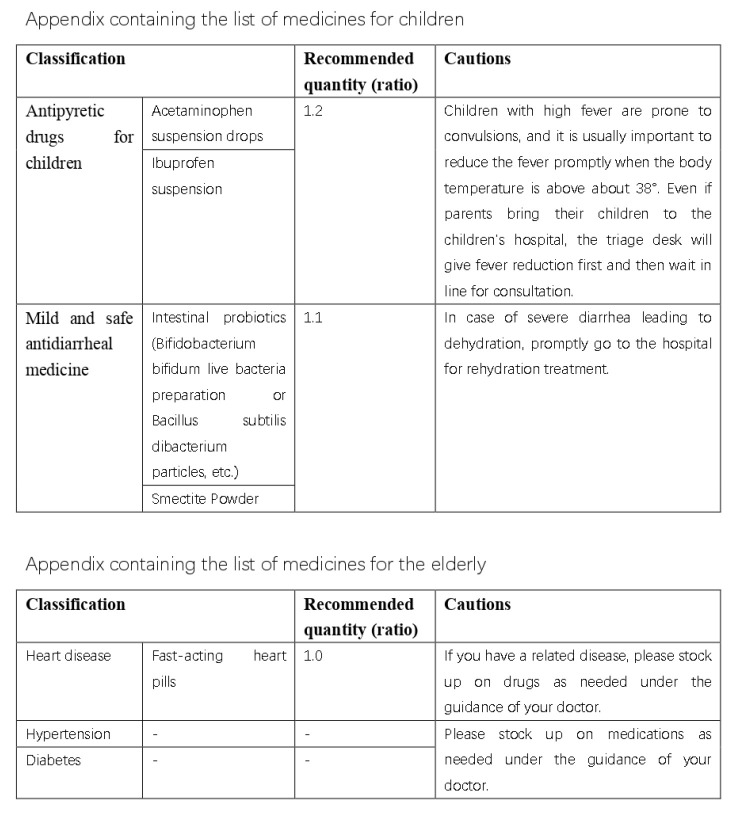
The initial version of the system.

Based on the results of our interviews and questionnaire research, we initially developed a practical domestic-medication system for the Chinese family, as shown in Figure 12, Figure 13, Figure 14 and Figure 15.

## 4. Validation and Iteration of Study Results

### 4.1. Verification Method: Interview with Senior Doctors

We obtained the effective questionnaire information integration, our form, which is divided into the general table and points table, general table for various situations of families that have applicability, and a schedule for different ages according to the number of families to supplement drug storage recommendations. Moreover, according to the recycling questionnaire, different areas of family use drugs in type and use frequency show no significant difference, so were not taken into account when sorting.

#### 4.1.1. Sample Selection

Based on our initial system, we sought out four senior physicians to evaluate it.

#### 4.1.2. Problem Setting

Finally, the following types of questions should change flexibly according to the doctor’s answer, such as which questions the doctor thinks has more reference value within the list.

#### 4.1.3. Participants

Four senior physicians with clinical experience were contacted as respondents.

(1) Setting up

Each interviewee received an introduction email that included the interview questions list and an information sheet (the description of interview aim, method, and the use of data). The email also linked to a self-booking system where the participant could easily select their interview time.

(2) Introduction

Each interview consisted of two personnel who are the interviewee and the interviewer (the researcher). The interviewer showed the information sheet and briefly summarized the interviewee’s study before the primary interview started.

(3) Agreement signature

A consent form was provided that presented eight relevant clauses about the agreement of participating in this study. Each interviewee was required to read and sign. Otherwise, the interview would not be continued.

(4) The main body of the interview

The interview followed four questions (Table 9). Each interview was audio-recorded with each interviewee’s permission.

#### 4.1.4. Data Collection and Analysis

We sorted and analyzed the doctors’ answers. (Table 10).

### 4.2. Validation Results

Based on the interview results of the interviewed doctors, our list became more practical and improved.

### 4.3. Optimize Iterations

In the validation part, the doctors suggested using amoxicillin, cephalosporin, and erythromycin to replace the acid drugs in tetracycline. Moreover, for the treatment of heart failure drugs, it was suggested that it is better to use nitroglycerin or compound Dans hen drop pill, a quick effect save heart pill. Based on these suggestions, we adjusted the content of our list.

### 4.4. Final Results

**Figure 16:**
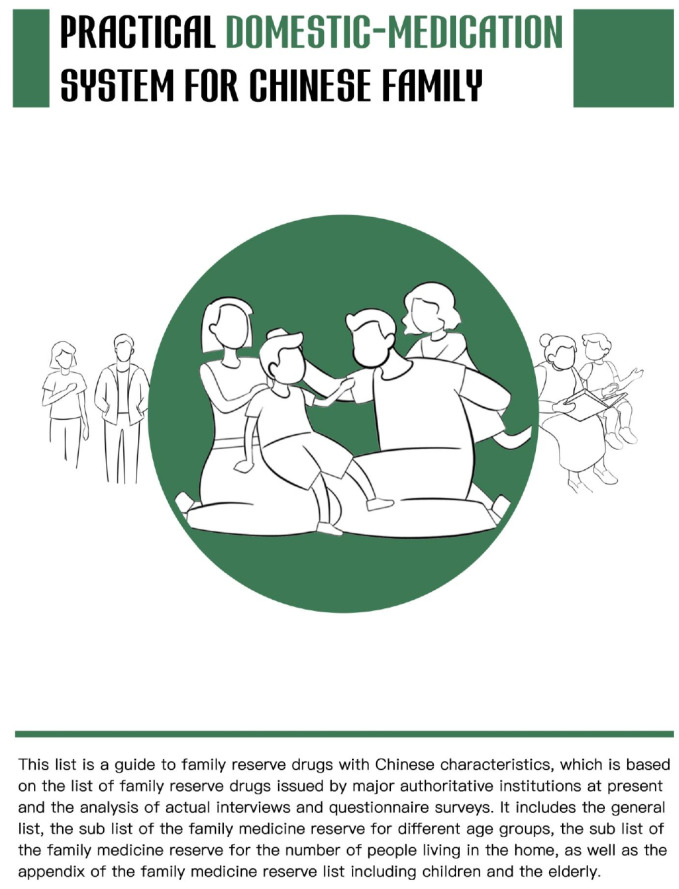
Final practical domestic-medication system.

**Figure 17:**
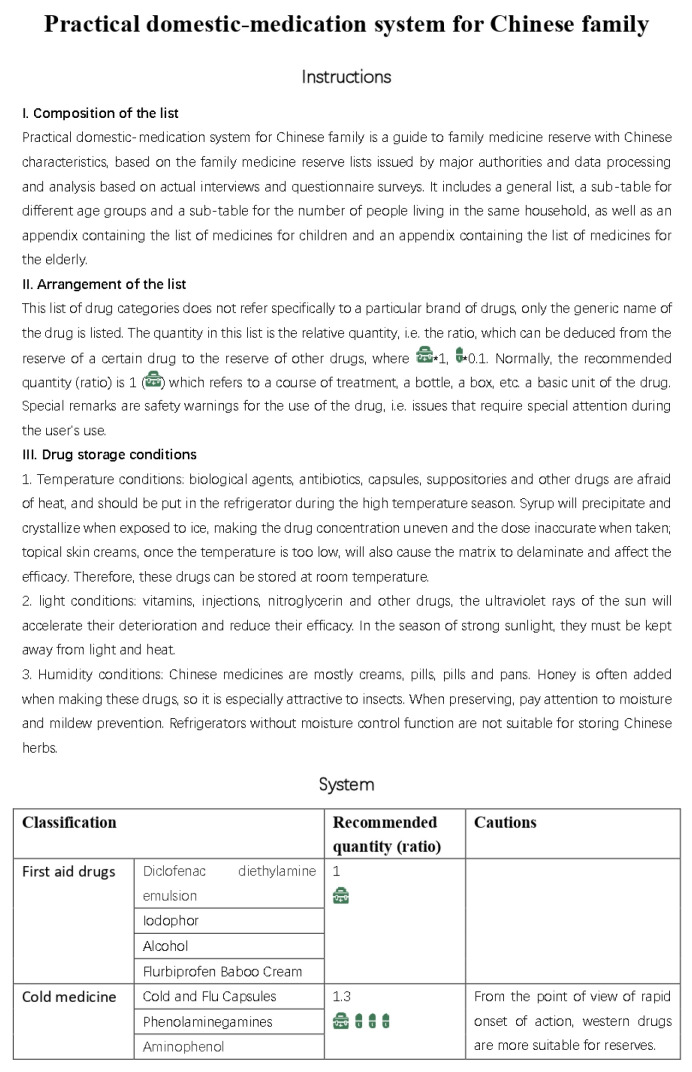
Final practical domestic-medication system.

**Figure 18:**
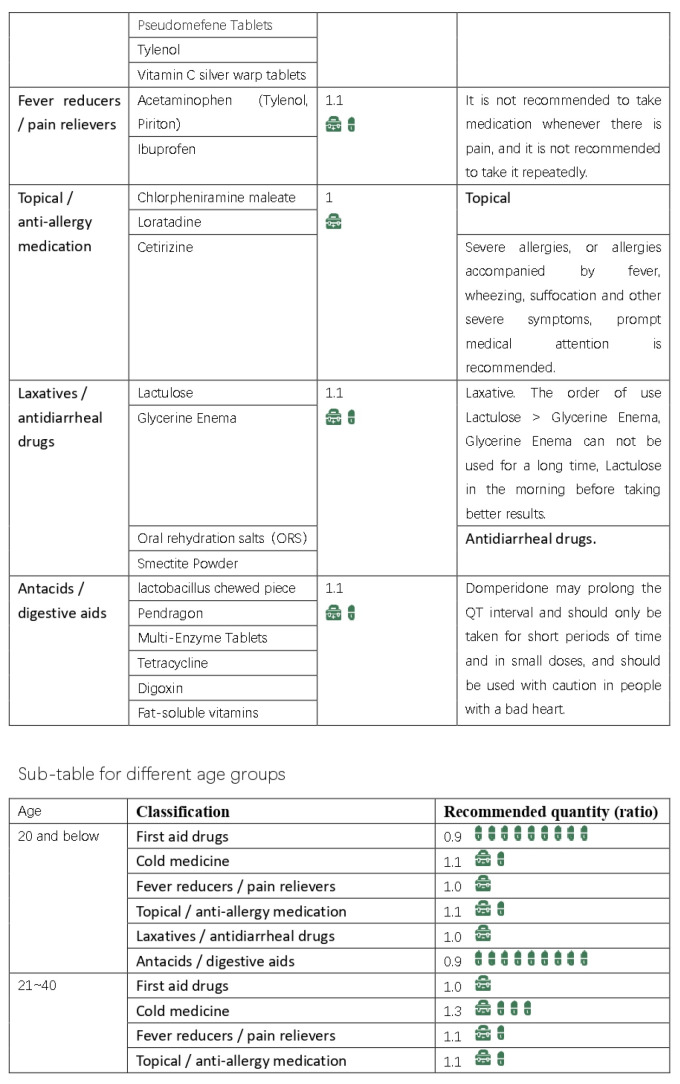
Final practical domestic-medication system.

**Figure 19:**
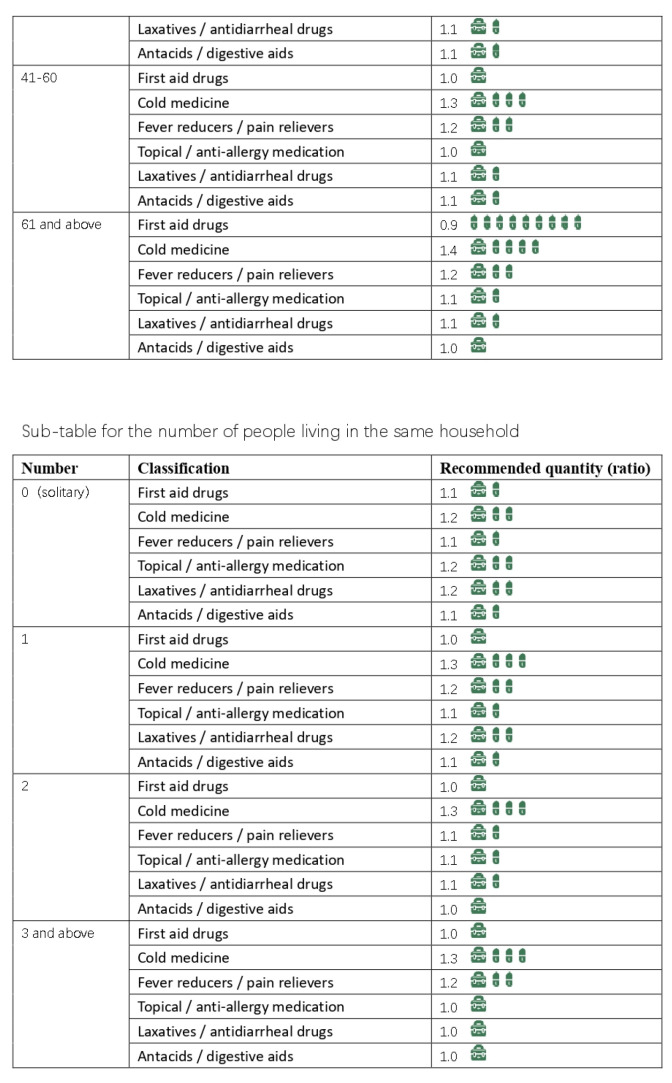
Final practical domestic-medication system.

**Figure 20:**
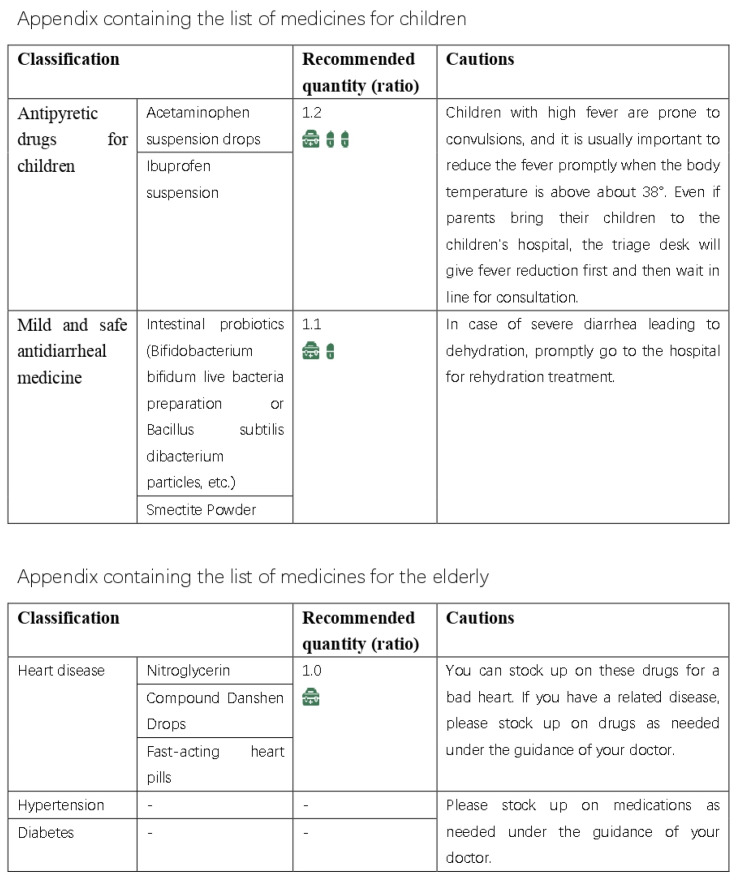
Final practical domestic-medication system.

Here are the final results, are shown in Figure 16, Figure 17, Figure 18, Figure 19 and Figure 20 (Supplementary File S1).

## 5. Discussion and Summary

In this paper, we focused on Chinese households’ stockpile of medicines. Based on the initial list of medicines, we determined the relationship between the number of different types of medicines and the relationship between the perceived importance of medicines and the frequency of using medicines through interviews and questionnaires and gradually improved the stockpile of medicines in Chinese households. Based on this, we established a standardized, systematic, and scientific system of household medicine stockpiling, which increased the connection between the type and quantity of medicine stockpile and actual life, reduced the misuse and waste of medicine caused by the expiration of medicine, and finally obtained the approval of doctors.

Due to the lack of attention to scientific stockpiling of medications, most respondents did not have a clear plan of stockpiling medications at home and lacked a general understanding, especially of the amount of stockpiling of different medications. Moreover, we found that people of different ages living together influenced the stockpiling of drugs, frequency of drug use, and perceived importance of drugs, but different regional factors had almost no influence on these issues. However, there is no correlation between the frequency of drug use and the importance of drugs, and there is a difference in the perception of drug stockpiling, with the actual frequency of use of drugs they consider important being low and the actual frequency of use of drugs they consider unimportant being high. All four senior doctors agreed with the existing problems we raised and the list we proposed and added to it, and finally, we formed a relatively complete list.

The innovation of our research results this time is to propose the relative stockpile amount of each kind of medicine in the process of household stockpiling. When a resident is familiar with the stockpile of a certain drug, they can quickly deduce the stockpile of other unfamiliar drugs through this list according to their age and family situation. This study makes the household stockpile list more relevant and can provide some reference for the general population to stockpile drugs so that the list can realistically solve the problems about the type and quantity of drugs stockpiled by residents in different living environments. It also helps families to stock a reasonable amount of medicines, thus reducing the risk of expired medicines being consumed by family members and the impact of expired medicines on the environment.

### Limitations

This system is only for the basic situation of Chinese residents, and it is our preliminary research on this issue. At the same time, we hope to conduct a study on the household stockpile in other regions in the future so that we can conclude a universal list of drugs worldwide.

## Figures and Tables

**Table 1:** Sample analysis.

Source	Classification	Advantages	Disadvantages
Popular science China	The composition of the family and recommendations for storing medicines for different age groups in the family	You can choose freely according to your family composition, flexible.	The classification of diseases for each age group is not accurate enough, which will lead to the problem of incomplete storage of drugs.
Dingxiang Doctor	The type of disease	Intuitive and straightforward to find the recommended medication by symptoms.	The classification of suitable use drugs for each age group is not clear, which may lead to drug abuse. The vague description of various types can only provide a simple reference. There are overlapping drugs in the classification.
PSM Pharmaceutical Shield Charity	The drug effect	You can know the effect and targeted disease of each drug treatment, to strengthen people’s cognition of disease classification.	The use of each age category is not clear.
Xinhuanet	The drug effect	Intuitive and straightforward to find the recommended medication by symptoms.	For each suitable age group, the use of drugs classification is not clear, the description of each type is vague and can only provide a simple reference, for each type of drug subdivision type does not include a more detailed explanation.

**Table 2:** Interview questions.

	Content
Question 1	How many people currently live with you?
Question 2	Is there a child living with you?
Question 3	Is there an old man living with you?
Question 4	Do you have the habit of seeing the shelf life of drugs?
Question 5	Do you have the habit of looking at drug storage conditions?
Question 6	Have you ever wanted to use a drug but you don’t have it in reserve at home?
Question 7	Have you ever had a situation where you want to use a drug but the drug in your home has expired?
Question 8	Which of the following drugs are in your store? First aid drugs (such as Diclofenac diethylamine cream, Iodophor, Alcohol, Yunnan Baiyao, etc.) Cold medicine Fever reducers (such as acetaminophen (Tylenin, Bentone)) pain relievers (ibuprofen, etc.) Topical (loratadine, cetirizine) Anti-allergy medication (chlorpheniramine maleate) Laxatives/antidiarrheal drugs (eg. lactulose, special, oral rehydration salts (ORS), montmorillonite powder, etc.) Antacids/digestive aids (such as stomach digestion tablets, risperidone, multienzyme tablets, tetracycline, digoxin, fat-soluble vitamins, etc.) Antipyretic drugs for children (such as acetaminophen suspension drops and ibuprofen) Mild and safe antidiarrheal medicine (enteroprobiotics (bifidobacterium preparations or B. subtilis particles, etc.), montmorillonite) Heart disease (nifedipine tablets, myocardial empowerment drugs, angiotensin receptor blockers) Blood pressure related drugs (angiotensin convertase inhibitors) Diabetes (oral hypoglycemic drugs (sulfonylureas, glinides, bigformides, thiazolidinediones, α glycosidase inhibitors, DDP-4 inhibitors, SGLT-2 inhibitors) injection agents (insulin, insulin analogs, and GLP-1 receptor agonists)) Hypoglycemia (grape powder, etc.) Rheumatic diseases Chronic respiratory diseases Other
Question 9	What is your frequency of drugs above?
Question 10	Please sort the necessity of the above drugs according to your life situation.
Question 11	What is the source of the list of your stored drugs?

**Table 3:** General family stockpile of drugs list.

Classification	Content
First aid drugs	Diclofenac diethylamine cream, Iodophor, Alcohol, Yunnan Baiyao
Cold medicine	
Fever reducers/pain relievers	Paracetamol, Ibuprofen
Topical/anti-allergy medication	Chlorpheniramine maleate, Loratadine, Cetirizine
Laxatives/antidiarrheal drugs	Lactulose, Glycerine Enema, Oral rehydration salts (ORS), Smectite Powder
Antacids/digestive aids	lactobacillus chewed piece, Pendragon, Multi-Enzyme Tablets, Tetracycline, Digoxin, Fat-soluble vitamins

**Table 4:** List of supplemental stockpile drugs for families with children.

Classification	Content
Antipyretic drugs for children	Acetaminophen suspension drops, Ibuprofen suspension
Mild and safe antidiarrheal medicine	Intestinal Probiotics (Bifidobacterium bifidum live bacteria preparation or Bacillus subtilis bacterium granules, etc.), Smectite Powder

**Table 5:** List of supplemental stockpile drugs for families with elderly members.

Classification	Content
Heart disease	Nifedipine Generic Tablets, Cardiac Enabler, Angiotensin II Receptor Blocker
Blood pressure-related drugs	Angiotensin-converting enzyme inhibitors
Diabetes	Oral hypoglycemic agents (sulfonylureas, glinides, biguanides, thiazolidinediones, alpha-glucosidase inhibitors, DDP-4 inhibitors, SGLT-2 inhibitors) Injectable preparations (insulin, insulin analogs, and GLP-1 receptor agonists)
Hypoglycemia	Glucose powder, etc.
Rheumatic diseases	
Chronic respiratory diseases	

**Table 6:** Summary and analysis of user interview results.

Questions	Answers	Analysis
*Q1 Have you ever wanted to use a certain medication but did not have it in stock at home?*	Very few people (1/10) have ever wanted to use medication but did not have it stocked at home, and based on current errand/flash delivery platforms, this is an easy problem to solve.	Respondents did not have a clear plan for stocking medications at home and lacked an overall understanding.
*Q2 Have you ever wanted to use a certain medication but the medication you have at home has expired?*	Almost all respondents (8/10) had experienced a situation where they wanted to use a certain medication but the medication stocked at home had expired, and these expired medications were often left over from the last illness they were cured of. When this happens more frequently, respondents are likely to develop the habit of regularly checking the shelf life of medications.	Not having the right idea of how much medicine to keep at home.
*Q3 Are there any medications on the reserve medication list?*	All respondents had cold medicines in stock, and most of them were Chinese medicines (Lotus Clear Capsules, wind-cold cold granules, anti-viral cold granules), whereas a few had western medicines (Neo control, cold spirit) in stock. Most of the respondents (7/10) stocked first-aid drugs such as iodine, Yunnan Baiyao, etc. Respondents with a large number of members and a complex family structure (including grandparents, parents, and children) or those who had lived in such families stocked relatively more types of drugs, and some of the drugs on the list were not currently stocked but had been stocked.	The list of drugs is strongly influenced by the composition of family members.
*Q4 How often are these drugs used?*	Cold medication was the most commonly used home stockpile according to the respondents, followed by emergency medication and laxatives/anti-diarrheal medication. This includes households with children, where respondents have a relatively clear understanding of the medications used at home.	Families without susceptible populations do not pay enough attention to drug use.
*Q5 What is the order of importance of these drugs?*	Cold and flu medication was the most important medication respondents felt they needed to stock up on, followed by antipyretics and emergency medication, then laxatives and antidiarrheal medication, and finally topical/anti-allergy medication and antacids/digestive aids, and this correlated with the frequency of illness. Because of frequent colds and the tendency to get a fever from colds, cold and fever reducers are often stocked together, and there is a correlation between the stockpiling of cold and fever reducers.	
*Q6 What is the reserve of these drugs?*	The majority of respondents who stockpile medication at home stockpile a course of appropriate medication, and a minority (1/5) buy a large amount of medication as a precaution.	
*Q7 Do you have a habit of looking at the storage conditions of drugs?*	More than half (6/10) of the respondents had the habit of checking the storage conditions of medications and related to the need for special storage of medications for the disease they were born with, after a special order from their doctor.	Insufficient attention to scientific stockpiling of drugs.
*Q8 Do you check the shelf life of your medication and regularly check the expiration date?*	All respondents were in the habit of checking the shelf life of their medications, especially before taking them, whereas only half of them (5/10) were in the habit of checking the shelf life of their medications regularly.	Not enough attention is paid to the shelf life of medicines stocked at home, and checking the shelf life only before taking them may be a safety hazard.
*Q9 What is the source of the drug list?*	The source of the respondents’ medication list was mainly what they knew about themselves and the advice of their doctors from past visits. This knowledge of oneself often comes from the medication stockpile of one’s family of origin, from which one’s children have learned the rules of family medication use and put them into practice in the family one has formed. At the same time, the type of medication is related to the type of illness, and the stockpile of medication for previous illnesses is relatively rich and complete, whereas there is a little stockpile of medication for illnesses that have not been suffered.	The drug list is derived from more subjective sources and lacks the support of objective medical advice.

**Table 7:** Model Summary ^a^.

Model	R	R^2^	Adjusted R^2^	Errors in Standard Estimation	Durbin Watson
1	0.756 ^b^	0.572	0.511	0.20840	1.788

^a^ Dependent variable: frequency of use; ^b^ Predictor variables: (constant), order of importance.

**Table 8:** Coefficient ^a^.

Model	Unstandardized Coefficient		Standardization Coefficient	T	Significance	Covariance Statistics	
	B	Standard Error	Beta			Tolerances	VIF
(Constants)	2.392	0.157		15.251	0.000		
Ranking of importance	0.105	0.034	0.756	3.060	0.018	1.000	1.000

^a^ Dependent variable: frequency of use.

**Table 9:** Interview questions.

Question	Content
Question 1	What is your opinion on drug storage at home?
Question 2	Can you take a look at these drug classification lists and select the drug storage classification list that you think is the most meaningful?
Question 3	Do you know what drug list will be more reference?

**Table 10:** Doctors’ answers.

Interviewee	Is It Necessary to Reserve Regular Drugs at Home	List of Recommended Drugs	Which Set of Lists Is Much Better	Better Reason
1	It is necessary to reserve some similar cold medicines, fever, and painkillers to relieve the patient’s condition. If timely treatment, can inhibit the disease and reduce unnecessary expenses. However, because 11 drugs are not allowed during the epidemic, some drugs may not have storage conditions.	I haven’t heard of it yet	Our comparison makes sense	The classification is more detailed, with a variety of conditions, such as age, being classified.
2	Regular family is necessary to store some drugs for an emergency, such as cold medicine, but may not reserve during the outbreak, need to reserve some similar cold medicine fever pain medicine to relieve the condition of the disease if timely treatment can inhibit illness, reduce unnecessary spending, but because the outbreak during 11 cases are not allowed to prescribe, so some drugs may not have storage conditions.	I haven’t heard of it yet	Our comparison makes sense	The age of the classification is more reasonable, children’s antipyretic is very necessary to store, adults with antipyretic, adults with dermatitis, and other drugs are necessary to store. But it is not necessary to age, 14 and 14 and the elderly can be classified. Remarks for all kinds of drugs are also more reasonable.
3	It is necessary, but antipyretic medicine or cold medicine is not allowed to be stored shortly, anti-allergy drugs can be stored, generally speaking, medical insurance reimbursement is not recommended to store, diarrhea medicine antidiarrhea medicine storage is of little significance.	I haven’t heard of it yet	Our comparison makes sense	It is best to do a schedule for the elderly and children, for the elderly over 60 and young children to do a form will add more reference value, but children are recommended to go directly to the hospital because there are no clear descriptions for children’s problems, parents may give children the wrong medicine
4	Yes, such as OTC	No research has been done	Each has its advantages	There is a clearer classification of external drugs, with more detailed medication-taking instructions, as well as contraindications

## Data Availability

The data presented in this study are available on request from the corresponding author.

## Supplementary Materials

The following are available online at https://www.mdpi.com/article/10.3390/ijerph20021060/s1, File S1: Final result_Practical Domestic-medication System for Chinese family.
