# Supplementary material for: Investigating and Developing a Practical Domestic-Medication System of Public Health for Chinese Family
Source: Int J Environ Res Public Health. 2023 Jan 6;20(2):1060. doi: 10.3390/ijerph20021060 (PMC9858686; doi:10.3390/ijerph20021060)
Supplement: Supplementary file 1 [file ijerph-20-01060-s001.zip › Final result_Practical Domestic-medication System for Chinese family.pdf]

PRACTICAL DOMESTIC-MEDICATION SYSTEM FOR CHINESE FAMILY

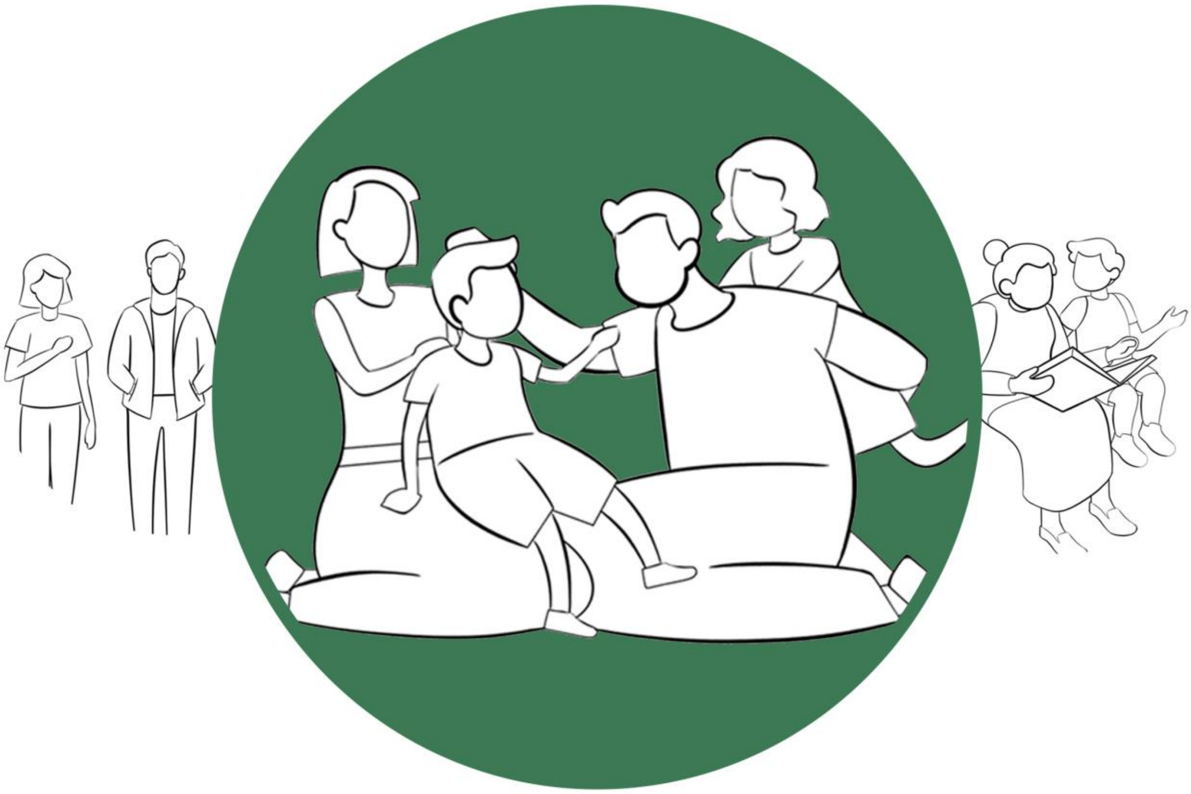

This list is a guide to family reserve drugs with Chinese characteristics, which is based on the list of family reserve drugs issued by major authoritative institutions at present and the analysis of actual interviews and questionnaire surveys. It includes the general list, the sub list of the family medicine reserve for different age groups, the sub list of the family medicine reserve for the number of people living in the home, as well as the appendix of the family medicine reserve list including children and the elderly.

Practical domestic-medication system for Chinese family

Instructions

I. Composition of the list

Practical domestic-medication system for Chinese family is a guide to family medicine reserve with Chinese characteristics, based on the family medicine reserve lists issued by major authorities and data processing and analysis based on actual interviews and questionnaire surveys. It includes a general list, a sub-table for different age groups and a sub-table for the number of people living in the same household, as well as an appendix containing the list of medicines for children and an appendix containing the list of medicines for the elderly.

II. Arrangement of the list

This list of drug categories does not refer specifically to a particular brand of drugs, only the generic name of the drug is listed. The quantity in this list is the relative quantity, i.e. the ratio, which can be deduced from the reserve of a certain drug to the reserve of other drugs, where *1, *0.1. Normally, the recommended quantity (ratio) is 1 () which refers to a course of treatment, a bottle, a box, etc. a basic unit of the drug. Special remarks are safety warnings for the use of the drug, i.e. issues that require special attention during the user's use.

III. Drug storage conditions

1. Temperature conditions: biological agents, antibiotics, capsules, suppositories and other drugs are afraid of heat, and should be put in the refrigerator during the high temperature season. Syrup will precipitate and crystallize when exposed to ice, making the drug concentration uneven and the dose inaccurate when taken; topical skin creams, once the temperature is too low, will also cause the matrix to delaminate and affect the efficacy. Therefore, these drugs can be stored at room temperature.
2. light conditions: vitamins, injections, nitroglycerin and other drugs, the ultraviolet rays of the sun will accelerate their deterioration and reduce their efficacy. In the season of strong sunlight, they must be kept away from light and heat.
3. Humidity conditions: Chinese medicines are mostly creams, pills, pills and pans. Honey is often added when making these drugs, so it is especially attractive to insects. When preserving, pay attention to moisture and mildew prevention. Refrigerators without moisture control function are not suitable for storing Chinese herbs.

System

**Classification**		**Recommended quantity (ratio)**	**Cautions**
First aid drugs	Diclofenac diethylamine emulsion	1	
	Iodophor		
	Alcohol		
	Flurbiprofen Baboo Cream		
Cold medicine	Cold and Flu Capsules	1.3	From the point of view of rapid onset of action, western drugs are more suitable for reserves.
	Phenolaminegamines		
	Aminophenol		

	Pseudomefene Tablets		
	Tylenol		
	Vitamin C silver warp tablets		
Fever reducers / pain relievers	Acetaminophen (Tylenol, Piriton)	1.1	It is not recommended to take medication whenever there is pain, and it is not recommended to take it repeatedly.
	Ibuprofen		
Topical / anti-allergy medication	Chlorpheniramine maleate	1	Topical
	Loratadine		
	Cetirizine		Severe allergies, or allergies accompanied by fever, wheezing, suffocation and other severe symptoms, prompt medical attention is recommended.
Laxatives / antidiarrheal drugs	Lactulose	1.1	Laxative. The order of use Lactulose > Glycerine Enema, Glycerine Enema can not be used for a long time, Lactulose in the morning before taking better results.
	Glycerine Enema		
	Oral rehydration salts（ORS）		Antidiarrheal drugs.
	Smectite Powder		
Antacids / digestive aids	lactobacillus chewed piece	1.1	Domperidone may prolong the QT interval and should only be taken for short periods of time and in small doses, and should be used with caution in people with a bad heart.
	Pendragon		
	Multi-Enzyme Tablets		
	Tetracycline		
	Digoxin		
	Fat-soluble vitamins		

Sub-table for different age groups

Age	**Classification**	**Recommended quantity (ratio)**
20 and below	First aid drugs	0.9
	Cold medicine	1.1
	Fever reducers / pain relievers	1.0
	Topical / anti-allergy medication	1.1
	Laxatives / antidiarrheal drugs	1.0
	Antacids / digestive aids	0.9
21~40	First aid drugs	1.0
	Cold medicine	1.3
	Fever reducers / pain relievers	1.1
	Topical / anti-allergy medication	1.1

	Laxatives / antidiarrheal drugs	1.1
	Antacids / digestive aids	1.1
41-60	First aid drugs	1.0
	Cold medicine	1.3
	Fever reducers / pain relievers	1.2
	Topical / anti-allergy medication	1.0
	Laxatives / antidiarrheal drugs	1.1
	Antacids / digestive aids	1.1
61 and above	First aid drugs	0.9
	Cold medicine	1.4
	Fever reducers / pain relievers	1.2
	Topical / anti-allergy medication	1.1
	Laxatives / antidiarrheal drugs	1.1
	Antacids / digestive aids	1.0

Sub-table for the number of people living in the same household

Number	Classification	Recommended quantity (ratio)
0 (solitary)	First aid drugs	1.1
	Cold medicine	1.2
	Fever reducers / pain relievers	1.1
	Topical / anti-allergy medication	1.2
	Laxatives / antidiarrheal drugs	1.2
	Antacids / digestive aids	1.1
1	First aid drugs	1.0
	Cold medicine	1.3
	Fever reducers / pain relievers	1.2
	Topical / anti-allergy medication	1.1
	Laxatives / antidiarrheal drugs	1.2
	Antacids / digestive aids	1.1
2	First aid drugs	1.0
	Cold medicine	1.3
	Fever reducers / pain relievers	1.1
	Topical / anti-allergy medication	1.1
	Laxatives / antidiarrheal drugs	1.1
	Antacids / digestive aids	1.0
3 and above	First aid drugs	1.0
	Cold medicine	1.3
	Fever reducers / pain relievers	1.2
	Topical / anti-allergy medication	1.0
	Laxatives / antidiarrheal drugs	1.0
	Antacids / digestive aids	1.0

Appendix containing the list of medicines for children

**Classification**		**Recommended quantity (ratio)**	**Cautions**
Antipyretic drugs for children	Acetaminophen suspension drops	1.2	Children with high fever are prone to convulsions, and it is usually important to reduce the fever promptly when the body temperature is above about 38°. Even if parents bring their children to the children's hospital, the triage desk will give fever reduction first and then wait in line for consultation.
	Ibuprofen suspension		
Mild and safe antidiarrheal medicine	Intestinal probiotics (Bifidobacterium bifidum live bacteria preparation or Bacillus subtilis dibacterium particles, etc.)	1.1	In case of severe diarrhea leading to dehydration, promptly go to the hospital for rehydration treatment.
	Smectite Powder		

Appendix containing the list of medicines for the elderly

**Classification**		**Recommended quantity (ratio)**	**Cautions**
Heart disease	Nitroglycerin	1.0	You can stock up on these drugs for a bad heart. If you have a related disease, please stock up on drugs as needed under the guidance of your doctor.
	Compound Danshen Drops		
	Fast-acting heart pills		
Hypertension	-	-	Please stock up on medications as needed under the guidance of your doctor.
Diabetes	-	-	
